# Recombinant production of a hard‐to‐express membrane‐bound cytochrome P450 in different yeasts—Comparison of physiology and productivity

**DOI:** 10.1002/yea.3441

**Published:** 2020-01-06

**Authors:** Johanna Hausjell, Dominik Schendl, Julia Weissensteiner, Christian Molitor, Heidi Halbwirth, Oliver Spadiut

**Affiliations:** ^1^ TU Wien Institute of Chemical, Environmental and Bioscience Engineering Gumpendorfer Straße 1a Vienna 1060 Austria

**Keywords:** chalcone 3‐hydroxylase, *Pichia pastoris*, *Saccharomyces cerevisiae*, yeast, physiology, recombinant protein production

## Abstract

Cytochrome P450s comprise one of the largest protein superfamilies. They occur in every kingdom of life and catalyse a variety of essential reactions. Their production is of utmost interest regarding biotransformation and structure‐function elucidation. However, they have proven hard to express due to their membrane anchor, their complex co‐factor requirements and their need for a redox‐partner. In our study, we investigated and compared different yeast strains for the production of the plant cytochrome P450 chalcone 3‐hydroxylase. To our knowledge, this is the first study evaluating different yeasts for the expression of this abundant and highly significant protein superfamily.

*Saccharomyces cerevisiae* and three different strains of *Pichia pastoris* expressing chalcone 3‐hydroxylase were cultivated in controlled bioreactor runs and evaluated regarding physiological parameters and expression levels of the cytochrome P450. Production differed significantly between the different strains and was found highest in the investigated *P. pastoris* MutS strain KM71H where 8 mg P450 per gram dry cell weight were detected. We believe that this host could be suitable for the expression of many eukaryotic, especially plant‐derived, cytochrome P450s as it combines high specific product yields together with straightforward cultivation techniques for achieving high biomass concentrations. Both factors greatly facilitate subsequent establishment of purification procedures for the cytochrome P450 and make the yeast strain an ideal platform for biotransformation as well.

## 1 INTRODUCTION

Cytochrome P450s, sometimes referred to as “Nature's most versatile catalysts” (Gillam, 2007) form one of the biggest protein superfamilies and are present in all kingdoms of life (Werck‐Reichhart & Feyereisen, 2000). They are responsible for the catalysis of uncountable reactions, ranging from detoxification over hormone synthesis to carbon source assimilation (Guengerich, Waterman, & Egli, 2016; Werck‐Reichhart & Feyereisen, 2000). The core of these enzymes consists of an iron porphyrin group, which classifies them as hemoproteins. Interestingly, aside from this characteristic feature cytochrome P450s often share only little homology regarding their amino acid sequences, sometimes below 16% (Werck‐Reichhart & Feyereisen, 2000). This makes recombinant expression of cytochrome P450s for structure‐function elucidation inevitable. Moreover, their recombinant production can be of interest for biotransformations, as these enzymes are able to specifically hydroxylate complex hydrocarbons (Girvan & Munro, 2016).

However, recombinant expression of eukaryotic cytochrome P450s is challenging due to a variety of reasons: (1) They have complex co‐factor requirements: Incorporation of the heme group into their core is essential for catalytic activity; (2) a redox partner protein, cytochrome P450 reductase, is required for catalytic activity; (3) eukaryotic cytochrome P450s are usually membrane bound, therefore they are either engineered as soluble enzyme and expressed in prokaryotic hosts as for instance *Escherichia coli* or they are natively expressed in eukaryotic hosts such as yeasts, mainly *Saccharomyces cerevisiae.* (Hausjell, Halbwirth, & Spadiut, 2018; Sudhamsu et al., 2010; Wagner et al., 2008).

In our study we wanted to recombinantly produce chalcone 3‐hydroxylase (CH3H), from *Dahlia variabilis*, a cytochrome P450 essential in the flavonoid pathway and responsible for yellow petal colorization and the formation of UV honeyguides (Harborne & Smith, 1987; Miosic et al., 2013; Schlangen, Miosic, Thill, & Halbwirth, 2010).

We wanted to express the cytochrome P450 as a full‐length protein for future structure elucidation. This seemed particularly interesting as only one cytochrome P450 has been crystallized including its native membrane anchor so far, although this region is not very well conserved (Monk et al., 2014; Nelson, 2006). As *E. coli* lacks inner organelles which makes most of the prokaryotic strains unsuitable for membrane protein production, we chose yeasts for CH3H expression (Rosano & Ceccarelli, 2014).

Interestingly, up to now, many more studies regarding cytochrome P450 expression have been conducted in *S. cerevisiae* rather than in *Pichia pastoris* (Hausjell, et al., 2018a). Therefore, we were curious if indeed baker's yeast is a superior host for recombinant production of these enzymes. We cultivated one *S. cerevisia*e strain and three different strains of *P. pastoris* (KM71H, GS115 and SMD1168H; Table 1) expressing CH3H in controlled bioreactor runs and analysed strain specific parameters as well as CH3H production levels. All three *P. pastoris* strains are frequently employed for recombinant protein expression (Ahmad, Hirz, Pichler, & Schwab, 2014). Strains KM71H and GS115 were chosen as they had been employed successfully in several cases for membrane protein production before (Byrne, 2015). However, most frequently strain SMD1163 was used as a host for the production of membrane proteins (Byrne, 2015). As this strain is no longer commercially available, we decided to investigate its close relative, strain SMD1168H, instead.

**Table 1:** **Genotypes of the investigated *P. pastoris* strains** Comparison of the different genotypes of the *P. pastoris* strains investigated for expression of CH3H. Abbreviations: *aox1*: alcohol oxidase 1; *arg4*: arginine requiring; *pep4*: vacuolar aspartyl protease deficient; *his 4*: histidine requiring

Strain	Genotype
KM71H	*aox1::ARG4,arg4*
SMD1168H	*pep4*
GS115	*his4*

KM71H is a methanol utilizer slow (MutS) strain carrying only the *alcohol oxidase 2* (*aox2)* gene. In contrast GS115 as well as SMD1168H both carry the *aox1* and *aox2* gene, characterizing them as methanol utilizer plus (Mut+) strains. SMD1168H is often favored for production of recombinant protein as it is protease deficient, avoiding degradation of target protein (Ahmad et al., 2014).

One study already investigated the expression of a membrane‐bound protein (catechol‐O‐methyltransferase) in a MutS and Mut+ strain, however, only in shake flasks, where the results strongly depended on the carbon source present (Pedro et al., 2015). We decided to shed more light on the suitability of Mut+ and MutS strains for membrane protein expression by performing controlled bioreactor runs of strains expressing the bitopic membrane protein CH3H.

To our knowledge, this is the first study comparing different yeasts as expression hosts for cytochrome P450s. In finding the most suitable expression host, our main criteria were high space‐time‐yields and high product titres, as we ultimately wanted to establish a purification procedure for the membrane bound cytochrome P450, which can be rather cumbersome as low recovery yields are an issue and stability can be challenging.

## 2 MATERIALS AND METHODS

### 2.1 Strains and transformation

#### 2.1.1 *S.* *cerevisiae*

**Figure A1:**
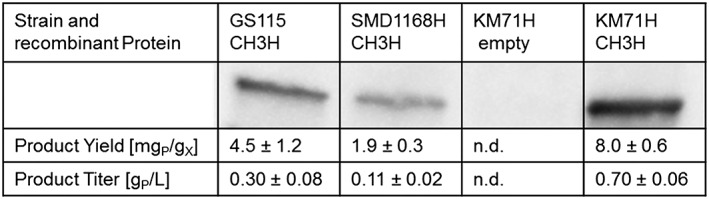
Additional file 1: Western Blot

The coding sequence of *Dv*CH3H was synthesized codon‐optimized for expression in yeast (Genscript, Piscataway, NJ, USA). The primers (Table A1. Additional file 1: Table of primers used for cloning) Figure A1. Additional file 1: TIFF, Western Blotwere designed by using the StarPrimer D´Signer software (IBA Lifesciences, Version 3.0.0.3). PR‐PCR was performed with the primer combination DvCH3H‐SF and DvCH3H‐SR as well as the Pfu DNA Polymerase (Thermo Scientific, Lithuania). StarGate® cloning and expression system (IBA Lifesciences, Goettingen, Germany) was used according to the manufacturer´s instructions. During the first cloning step, the PR‐PCR product was inserted into pENTRY‐IBA51 to generate the donor vector. In the second step, the insert of the donor vector was subcloned into the acceptor vector pYSG‐IBA103. This vector encodes a C‐terminal Twin‐Strep‐Tag® (tandem peptide WSHPQFEK with an internal linker region). The purified plasmid DvCH3H pYSG‐IBA103 was transformed into the *S. cerevisiae* INVSc1 by using the S.c. Easy CompTM Transformation Kit (Invitrogen, Carlsbad, USA).

#### 2.1.2 *P.* *pastoris*

Strains KM71H, SMD1168H and GS115 were purchased as stab cultures from Thermo Fisher (Waltham, MA, USA). pPICZ A plasmids were purchased from Invitrogen (Carlsbad, CA, USA). The gene coding for *Dv*CH3H was synthesized with the recognition sites for the endonucleases EcoRI and NotI, the Kozak consensus sequence as well as codon‐optimized for expression in yeast (Genscript, Piscataway, NJ, USA).

The vector pPICZ A (Invitrogen, Carlsbad, USA) was modified. The C‐terminal myc‐epitope was exchanged with the TEV cleavage site (GAGAACCTCTACTTCCAATCG). This mutation was generated directly from the pPICZ A vector using the Q5 Site‐Directed Mutagenesis Kit (NewEngland Biolabs, Vienna, Austria). Primers (Table A1. Additional file 1: Table of primers used for cloning) were designed with the online tool NEBaseChanger provided at http://nebasechanger.neb.com.

The genes were cloned by restriction digest with EcoRI and NotI (New England Biolabs, Vienna, Austria) into the modified pPICZ A with TEV cleavage site to form the corresponding *Dv*CH3H pPICZ A construct. The plasmid was transferred into the competent *E. coli* Top 10 cells (IBA Lifesciences, Goettingen, Germany) by heat shock.

The integrity of the constructs was confirmed by commercial sequencing (Microsynth Austria AG, Vienna, Austria).

*P. pastoris* strains were then transformed according to Easy Select ^TM^ *Pichia* Expression Kit (Invitrogen, Thermo Scientific, Waltham, MA, USA). To check successful integration of CH3H, the yeast strains were grown on BMGY media and BMMY media. Expression of target protein was confirmed by western blotting (see below).

### 2.2 Cultivations

All cultivations consisted of a batch and fed‐batch phase for biomass generation, followed by an induction phase for production of target protein. Samples were taken in the beginning of the batch, the end of the batch and start of the fed‐batch, before induction and every 24 h during induction. All samples were analysed regarding dry cell weight as well as metabolites in the supernatant.

#### 2.2.1 *S.* *cerevisiae*

##### Preculture

500 mL of SGI Media supplemented with 0.02 g α‐L‐Tyrpthophan were inoculated with frozen stocks (7.5 ml, ‐80 °C) and shaken at 30 °C and 230 rpm overnight in an Infors HR Multitron shaker (Infors, Bottmingen, Switzerland).

##### Batch and fed‐batch phase

The cultivation was carried out in an InforsHT Labfors 5 fermenter equipped with a 5 L glass‐vessel (Infors, Bottmingen, Switzerland). All process parameters were logged at least every 30 s and controlled by the process information management system Lucullus (Securecell, Schlieren, Switzerland). 1.8 L Delft Media (Jensen et al., 2014) with 20 g/L glucose (supplemented with 0.002 g α‐L‐Tyrpthophan and 0.002 α‐L‐Histidine per gram of glucose) were inoculated with 200 mL preculture. The temperature was kept constant at 30 °C, the reactor was stirred at 1400 rpm and aerated at 2 vvm to avoid oxygen limitation. dO was monitored with a fluorescence dissolved oxygen electrode Visiferm DO425 (Hamilton, Reno, NV, USA). pH was measured with an EasyFerm electrode (Hamilton, Reno, NV, USA) and kept constant at 6.0 by the addition of NH_4_OH. Addition of base was recorded gravimetrically. CO_2_ and O_2_ in the exhaust gas were analysed by a Marino Müller gas analyser (Marino Mueller, Egg, Switzerland). The batch phase lasted 26 hours, indicated by a drop in the CO_2_ signal and an increase in dissolved oxygen and yielded 1.5 g/L biomass. Thereafter a fed‐batch phase was started at a constant q_s,glu_ using feed forward strategy according to equation 1 with F being the feed rate in g/h, q_s_ being the specific substrate uptake rate in g/g/h, X being the biomass concentration in g/L, V being the reactor volume in L and W being the amount of substrate per gram of feed in g/g.

(1) $$F=q_{s}*X*\frac{V}{W}\;$$

We fed with a 450 g/L glucose feed (supplemented with 0.002 g α‐L‐Trypthophan and 0.002 α‐L‐Histidine per gram of glucose) at a q_s,glu_ of 0.4 g/g/h for 48 h reaching a biomass concentration of 10 g/L. The feed was pumped through a preciflow peristaltic pump (Lambda Laboratory Instruments, Brno, Czech Republic) controlled by the process information management system and its addition was monitored gravimetrically.

##### Induction phase

Induction was performed by addition of copper sulfate to a final concentration of 0.5 mM. In addition, a pulse of hemin‐chloride to a final concentration of 5 μM was added. The feed‐rate was turned down from 0.4 to 0.05 g/g/h to avoid stressing the cells. Induction for production of target protein lasted 120 h.

#### 2.2.2 *P.* *pastoris*

##### Preculture

Frozen stocks (1.5 mL, ‐80 °C) were cultivated in 100 mL of YNB media (Carl Roth, Germany) in 1000 mL shake flasks at 30 °C and 230 rpm overnight in an Infors HR Multitron shaker (Infors, Bottmingen, Switzerland).

##### Batch and fed‐batch phase

100 mL of the preculture were transferred aseptically to the respective culture vessel containing 900 mL BSM medium (Invitrogen, Thermo Scientific, Waltham, MA, USA) with 60 g/L glycerol, supplemented with trace element solution (4.35 mL/L PTM1) and for strain GS115 0.002 α‐L‐Histidine per gram of glycerol. Batch cultivations were carried out in 2.7 L glass vessels (DAS‐Gip parallel bioreactor systems, Eppendorf, Hamburg, Germany) at 30 °C and a fixed agitation speed of 1400 rpm. pH was monitored with the EasyFerm Plus pH sensor (Hamilton, Reno, NV, USA) and kept constant at 5.0 by addition of 12.5 % NH_4_OH, the amount being monitored by DAS‐Gip MP8 Multi pump module (Eppendorf, Hamburg, Germany). The culture was aerated with 2 vvm with a mixture of pressurized air and pure oxygen. The ratio of air to oxygen was adjusted in a way that the dissolved oxygen (dO) was kept above 30%. The dO was measured with a fluorescence dissolved oxygen electrode Visiferm DO425 (Hamilton, Reno, NV, USA). Off‐gas (CO_2_ and O_2_) was measured with a DAS‐Gip GA gas analyser (Eppendorf, Hamburg, Germany). Temperature, pH, dO, pump rates (feed and base), inlet gas flow as well as CO_2_ and O_2_ in the off‐gas were measured online and logged every 30 s into the process information management system (DASware control: Eppendorf, Hamburg, Germany). The end of the batch phase and therefore the complete consumption of the substrate glycerol was indicated by an increase of dO and a drop in the CO_2_ exhaust gas signal. It resulted in 30 g/L dry cell mass. After the batch phase the glycerol feed (500g/L glycerol, 12mL/L PTM1, 0.3 mL/L Struktol J650 and in case of strain GS115 also 0.002 α‐L‐Histidine per gram of glycerol) was started at a constant q_s,gly_ of 0.08 g/g/h for 5 h, reaching biomass concentrations of 80 g/L. The feed‐rates were adjusted using a soft‐sensor tool (Wechselberger, Sagmeister, & Herwig*,* 2013).

##### Induction phase

For induction, a methanol adaption pulse (pure methanol supplemented with 12 mL/L PTM1) was added to the cultivations reaching a final concentration of 0.5 % (v/v) in the bioreactor. In addition, a pulse of hemin‐chloride to a final concentration of 5 μM was added. After complete metabolization of the first pulse, a second pulse of 1 % (v/v) was added for determination of the maximum specific methanol uptake rate (q_s,meoh,max_). Thereafter a methanol feed (400 g/L Methanol, 4 mL/L PTM1, 0.3 mL/L Struktol J650) was started. We fed at a constant q_s,meoh_ of 0.03 g/g/h until an overall induction time (starting with the adaption pulse) of 120 h was reached.

#### 2.2.3 Analysis of dry cell weight and metabolites

Dry cell weight, optical density at 600 nm (OD_600_) as well as metabolite concentrations in the supernatant were analysed offline during the cultivation. For analysis of the cell dry weight, 1 mL culture broth was transferred into pre‐dried and pre‐weighed 2 mL plastic tubes and centrifuged (4500 g, 4 °C, 10 min). The supernatants were collected and frozen for subsequent HPLC analysis of metabolites. The pellet was washed twice with 1 mL 0.9 % NaCl solution before drying for 72 hours at 105 °C.

Metabolites in the supernatant were analysed via HPLC (Thermo Scientific, Waltham, MA, USA) on an Aminex column (Bio‐Rad, Vienna, Austria) at a constant flow of 0.6 ml/min at 60 °C. The mobile phase consisted of 4 mM H_2_SO_4_ and sugars were detected with a Shodex RI‐101 refractive index detector (DataApex, Prague, Czech Republic). Analysis of the chromatograms was performed using Chromeleon Software (Dionex, Sunnyvale, California, USA).

### 2.3 Analysis of CH3H expression levels

The cells were harvested and resuspended in 200 mM potassium phosphate buffer pH 8.0, 500 mM NaCl to a biomass concentration of 50 g/L. Cell disruption was performed by high pressure homogenization for 10 passages at 1500 bar with a PandaPlus 2000 (GEA, Düsseldorf, Germany).

The homogenized samples as well as a His‐6‐ tagged protein (flavanone 3‐hydroxylase FHT (Hausjell, Weissensteiner, Molitor, Halbwirth, & Spadiut, 2018) with known concentration, used as standard, were incubated 1:1 with a 2x concentrated Lämmli buffer (Laemmli, 1970) at 100 °C for 15 minutes. In case of the strep‐tagged CH3H expressed by *S. cerevisiae* a commercial standard (Precision Plus Protein™ Unstained Protein Standards, Strep‐tagged, Biorad, Vienna, Austria) was used. 5 μL of samples and dilutions of standards were loaded onto an Any kD™ Mini‐PROTEAN® TGX™ Precast Protein Gel, 10‐well, 30 μL (Bio‐Rad, Vienna, Austria). Gels were run in SDS buffer in a Mini‐PROTEAN® Tetra Vertical Electrophoresis Cell (Bio‐Rad, Hercules, CA, USA) at a constant voltage of 180 V for 35 min.

The gel was blotted with 350 mA for 100 minutes onto a nitrocellulose blotting membrane (Amersham™Protran™0.2μm NC, GE, Boston, Massachusetts, USA). The membrane was blocked (ROTI®‐BLOCK, Carl Roth, Germany) at 4°C over night. After washing, the membrane was incubated with an Anti‐His‐HRP antibody (MACS Miltenyi Biotec, Germany) for 2 hours, washed again and incubated 5 min with SuperSignal™ West Pico PLUS Chemiluminescent Substrate (Thermo Scientific, Waltham, MA, USA). Gel Doc XR system and ImageLab software (Bio‐Rad, Hercules, CA, USA) were used for detection and quantification.

### 2.4 Data analysis

For determination of statistical significance of differences between results, non‐parametric Kruskal‐Wallis tests were performed due to the small sample (usually three measurements). Significance of differences was determined at a 95 % confidence level.

## 3 RESULTS AND DISCUSSION

### 3.1 Cultivations of *S. cerevisiae*

We first investigated expression of CH3H in *S. cerevisiae* as, in general, higher cytochrome P450 expression titres were found compared with studies in *P. pastoris.* However, to our knowledge there is no study, where the same cytochrome P450 has been expressed in both hosts, which makes comparisons difficult (Hausjell, Halbwirth, & Spadiut, 2018). Strain INVSc1 was chosen together with the CUP1 promoter system, where production of target protein is induced upon addition of copper (Butt & Ecker, 1987). The yeast cells were first cultivated in batch and fed‐batch mode for biomass generation and subsequently induced by the addition of copper sulfate to a final concentration of 0.5 mM.

#### 3.1.1 Physiological parameters

Physiological parameters were calculated and are summarized in Table 2.

**Table 2:** **Physiological parameters of *S. cerevisiae* INVSc1 expressing CH3H** Physiological parameters of *S. cerevisiae* INVSc1 expressing CH3H during a cultivation consisting of a batch and fed‐batch phase followed by an induced fed‐batch. Standard deviations were calculated from triple measurements and calculated by error propagation for rates and yields. Abbreviations: q_s,Glu_: specific glucose uptake rate; q_P, EtOH_: specific ethanol production rate; q_P,Gly_: specific glycerol production rate; μ: specific growth rate; Y_X/Glu_: biomass yield on glucose; Y_CO2/Glu_: CO_2_ yield on glucose; Y_EtOH/Glu_: ethanol yields on glucose; Y_Gly/Glu_: glycerol yield on glucose; C‐balance: carbon balance

Phase	q_s,Glu_ [g_s_/g_x_/h]	q_P, EtOH_ [g_s_/g_x_/h]	q_P,Gly_ [g_s_/g_x_/h]	μ [h^‐1^]	Y_X/Glu_ [C‐mol/C‐mol]	Y_CO2/Glu_ [C‐mol/C‐mol]	Y_EtOH/Glu_ [C‐mol/C‐mol]	Y_Gly/Glu_ [C‐mol/C‐mol]	C‐balance [‐]
Batch	1.021 ± 0.15	0.338 ± 0.04	0.193 ± 0.08	0.074 ± 0.007	0.08 ± 0.005	0.35 ± 0.02	0.42 ± 0.02	0.08 ± 0.02	0.92 ± 0.03
Fed‐batch	0.412 ± 0.08	0.085 ± 0.07	0.057 ± 0.05	0.040 ± 0.01	0.07 ± 0.003	0.33 ± 0.02	0.57 ± 0.07	0.08 ± 0.02	1.06 ± 0.1
Induced fed‐batch	0.047 ± 0.003	0.012 ± 0.0007	0.009 ± 0.0005	0.005 ± 0.0001	0.09 ± 0.04	0.40 ± 0.07	0.35 ± 0.02	0.02 ± 0.003	0.86 ± 0.1

The strain was cultivated on glucose as source of carbon, as there was no growth detectable when glycerol was used as C‐source in a defined medium (data not shown).

In the batch phase, high ethanol and glycerol production (up to 7 g/L and 2 g/L, respectively) were observed, coming hand in hand with a low growth rate and low biomass yield. Larsson et al. found similar values before for glucose batches of the INVSc1 strain, when they used it for recombinant laccase expression (Larsson, Cassland, & Jonsson, 2001). The high ethanol formation is a result of the well‐known Crabtree effect, which occurs in *S. cerevisiae* whenever glucose is present in excess (Ghosh & De, 2017; Pfeiffer & Morley, 2014; Wojtczak, 1996). However, even during the uninduced fed batch, where glucose was fed in limiting amounts, ethanol and glycerol were produced, although at a lower rate. This nevertheless resulted in concentrations of up to 39 g/L and 12 g/L, respectively. Due to this vast production of metabolites the biomass never exceeded levels of 14.6 g/L dry cell mass. Moreover, it has been reported that concentrations of 4‐6 v/v % or 32‐47 g/L ethanol strongly induce stress response in the cells, similar to a heat shock response (Piper, 1995) and it has also been shown that viability decreased below 80 % within one hour after a 5 v/v % (or 39 g/L) pulse of ethanol (Birch & Walker, 2000). None of these factors seemed ideal, however, we still wanted to investigate expression of CH3H.

#### 3.1.2 Production of cytochrome P450

Productivity was investigated on a western blot, with strep‐tagged protein standards, where a specific product yield of 0.7 ± 0.1 mg CH3H per gram biomass was found, which, at a biomass concentration of 14.6 g/L, corresponds to a volumetric titre of 9.6 ± 1.9 mg CH3H per litre of cultivation broth. In literature the highest values for expression of P450s in yeast range around 50‐400 pmol/mg protein (Hausjell, Halbwirth, & Spadiut, 2018). In comparison, the titre achieved for CH3H expression in *S. cerevisiae* (53 pmol/mg protein) seemed to be at the lower end and was perhaps at least partially a result of the high stress put on the cells by the high ethanol levels.

All of these results led us to conclude that *S. cerevisiae* was not a suitable host for the production of CH3H.

### 3.2 Cultivations of *P. pastoris* KM71H, GS115, SMD1168H

Three different *P. pastoris* strains (KM71H, GS115, SMD1168H; Table 1) were transformed for expression of the plant P450 under control of the alcohol oxidase 1 (AOX1) promoter. All were cultivated analogously and to investigate effects from recombinant protein production, also an empty strain (“KM71H empty”) was fermented identically: A batch was carried out on glycerol for biomass generation followed by a glycerol fed‐batch with the same purpose. Thereafter methanol was pulsed to allow the cells to adapt to the different C‐source. A second pulse of this substrate was applied afterwards allowing determination of the maximum methanol uptake rate q_s,meoh,max_, similar to previous studies from our group (Dietzsch, Spadiut, & Herwig, 2011a; Dietzsch, Spadiut, & Herwig, 2011b). Subsequently a methanol fed‐batch was started to induce production of CH3H.

#### 3.2.1 Physiological parameters

**Figure 1:**
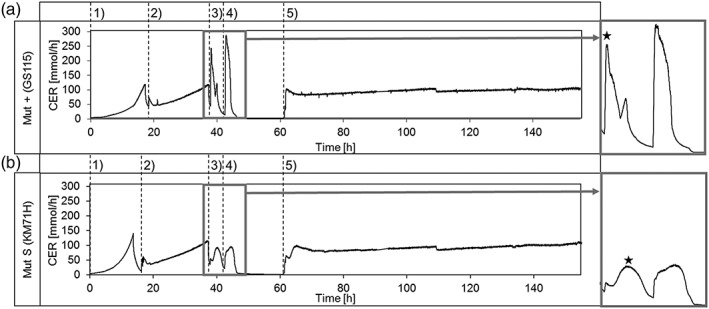
**Carbon dioxide evolution rate (CER) over time during the cultivation of Mut+ strain GS115 (A) and MutS strain KM71H (B).** The CER during the methanol pulses is zoomed out (right). The adaption time was calculated from addition of the pulse until the maximum of the CER was reached, indicated by the star. The different phases during the cultivation are numbered chronologically: The glycerol batch phase (1) was followed by a glycerol fed‐batch (2). Thereafter, the adaption pulse of 0.5 % methanol was added (3) and a second methanol pulse with 1 % was applied after complete metabolization of the first (4). After that, a methanol fed‐batch was started (5) which lasted for 72 h

All four strains were investigated regarding characteristic physiological parameters during the cultivation. The carbon dioxide evolution rate (CER) was calculated and is plotted over time in Figure 1 exemplarily for one MutS and one Mut+ strain. The most striking difference occurs during the methanol pulses: The CER in the MutS strain KM71H rises much slower compared with GS115 and SMD1168H. The first step in methanol utilization is conversion to formaldehyde and hydrogen peroxide by AOX1 and/or AOX2. This step happens faster in Mut+ strains as they harbor both AOX1 and AOX2. After conversion to formaldehyde, CO_2_ is formed in the dissimilative branch of the MUT pathway, which can also happen faster if formaldehyde conversion is elevated. Therefore, as expected, the CER can rise quicker in the Mut+ strains. The CER in all strains shows a characteristic double‐peak during the methanol pulses. This is also a result from the MUT pathway, caused by the formation of toxic hydrogen peroxide in the initial step (Lin‐Cereghino et al., 2006; Macauley‐Patrick, Fazenda, McNeil, & Harvey, 2005).

Based on the CER, biomass and substrate concentration, strain specific parameters during the cultivation were calculated and are summarized in Table 3.

**Table 3:** **Physiological parameters for the *P. pastoris* strains.** Physiological parameters of three *P. pastoris* strains expressing CH3H as well as one empty strain. Standard deviations were calculated from triple measurements and calculated by error propagation. Differences in the maximum specific methanol uptake rates were determined significant between Mut+ and MutS strains at a confidence level of 95 %. In the SMD1168H cultivation strong methanol accumulation occurred after 48 h of induction. Therefore, the feed‐rate was reduced. However, the biomass concentration continuously decreased as a result of feeding close to the maintenance level. This resulted in an extremely low biomass yield and high standard deviation thereof. A star in the table marks respective parameters. Abbrevations: μ_max,gly_: maximum growth rate on glycerol; q_s,max,gly_: maximum specific glycerol uptake rate; Y_X/S_: biomass yield on the respective substrate Y_CO2/S_: CO_2_ yield on the respective substrate; C‐Balance: carbon balance; t_adapt_: adaption time to methanol; q_s,adapt_: specific methanol uptake rate during adaption time; q_s,max,meoh_: maximum methanol uptake rate

	Phase	Parameter	GS115 CH3H	SMD1168H CH3H	KM71H CH3H	KM71H empty
Gly	Batch	μ_max,gly_ [h^‐1^]	0.21 ± 0.02	0.25 ± 0.06	0.29 ± 0.02	0.26 ± 0.02
		q_s,max,gly_ [g_s_/g_x_/h]	0.38 ± 0.03	0.45 ± 0.11	0.54 ± 0.03	0.47 ± 0.03
	Fed‐batch	Y_X/S_ [C‐mol/C‐mol]	0.74 ± 0.05	0.75 ± 0.16	0.63 ± 0.06	0.67 ± 0.02
		Y_CO2/S_ [C‐mol/C‐mol]	0.33 ± 0.03	0.33 ± 0.02	0.32 ± 0.06	0.32 ± 0.05
		C‐Balance[‐]	1.07 ± 0.07	1.08 ± 0.14	0.95 ± 0.12	0.99 ± 0.03
MeOH	0.5 % Pulse	t_adapt_ [h]	0.22	0.26	2.25	2.38
		q_s,adapt_ [g_s_/g_x_/h]	0.024 ± 0.002	0.028 ± 0.002	0.029 ± 0.002	0.027 ± 0.002
	1 % Pulse	q_s,max,meoh_ [g_s_/g_x_/h]	0.066 ± 0.005	0.072 ± 0.004	0.039 ± 0.002	0.054 ± 0.004
	Fed‐batch	Y_X/S_ [C‐mol/C‐mol]	0.16 ± 0.06	0.02 ± 0.29*	0.39 ± 0.08	0.38 ± 0.03
		Y_CO2/S_ [C‐mol/C‐mol]	0.87 ± 0.07	0.99 ± 0.25*	0.80 ± 0.04	0.77 ± 0.09
		C‐Balance[‐]	1.03 ± 0.11	1.02 ± 0.21*	1.19 ± 0.10	1.15 ± 0.11

While on glycerol all strains show similar behaviour, there are striking differences once they are grown on methanol. The adaption time to methanol (time from pulse addition to the maximum of the CER) differs strongly when comparing KM71H with SMD1168H and GS115, the one in KM71H being approximately by a factor ten longer. This again results from the differences in the methanol utilization of the strains. It goes hand in hand with the maxima of the specific methanol uptake rates, where the Mut+ strains GS115 and SMD1168H exhibit higher values than the MutS strain. As they have two AOX enzymes, they can convert more methanol per biomass per hour. When comparing the empty KM71H strain with the producing one, it can be stated that the maximum methanol uptake rate is slightly lower in the producing one. We believe this is a result from the additional burden of recombinant expression of the target protein. The same was found before by our group when a native KM71H strain was compared with a horseradish‐peroxidase expressing one (Dietzsch et al., 2011b).

#### 3.2.2 Production of cytochrome P450

Motivated by the promising results of the cultivations of the different *P. pastoris* strains, we investigated whether we could detect differences in recombinant protein production in the studied strains. Indeed, we found striking differences in specific product titres when we investigated CH3H expression on western blots (Figure A1. Additional file 1: TIFF, Western Blot).

Strain KM71H clearly produced the most target protein with 8.0 ± 0.6 mg_P_/g_X_, followed by GS115 (4.5 ± 1.2 mg_P_/g_X_) and SMD1168H (1.9 ± 0.3 mg_P_/g_X_). It is the only MutS strain among the investigated and we have two hypotheses for this behaviour. KM71H in contrast to GS115 and SMD1168H only expresses AOX2 when fed with methanol but not AOX1. We believe that this results in less stress for the organism, as for the other strains also AOX1 has to be expressed at high levels. It has been reported that when grown on methanol, AOX can make up 20‐30% of total protein (Zhang et al., 2009). This could make a crucial difference especially during the production of hard‐to‐express proteins such as the membrane bound CH3H. Another difference between Mut+ and MutS strains is the hydrogen peroxide concentration during methanol metabolization, which is elevated in Mut+ strains. Hydrogen peroxide is deleterious to cytochrome P450s (Guengerich, Martin, Sohl, & Cheng, 2009), which is why MutS strains might be more suitable for their production.

Among the Mut+ strains, GS115 outcompeted SMD1168H in regard to CH3H production. This was most likely due to the fact that in the cultivation of strain SMD1168H methanol accumulation was observed. Although we only fed at a specific methanol uptake rate of 0.03 g/g/h, which is below half of the maximum specific uptake rate, we observed methanol accumulation 48 h after starting the methanol fed‐batch. We turned down the feed rate to 0.01 g/g/h, however, such low feed rates only deliver energy close the maintenance level which results in neither growth nor productivity (Capone, Horvat, Herwig, & Spadiut, 2015). We hypothesize that the methanol accumulation in strain SMD1168H could be caused by its vacuolar aspartyl protease deficiency (Table 1). It has previously been shown that yeasts lacking this gene show increased apoptosis in the presence of hydrogen peroxide (Alugoju, Janardhanshetty, Subaramanian, Periyasamy, & Dyavaiah, 2018), which is produced during the first step of methanol metabolization.

Within this study we investigated expression of CH3H on a methanol‐only feed in the three different *P. pastoris* strains. Another option instead of feeding methanol alone would be the application of mixed feeds of methanol together with glycerol or other C‐sources. This has especially often been applied for MutS strains, as they grow slower on methanol (Krainer et al., 2012). For Mut+ strains studies with mixed feeds showed the same or even lower productivity (Jahic, Veide, Charoenrat, Teeri, & Enfors, 2006; Jungo, Marison, & von Stockar, 2007). This led us to conclude that such process conditions could potentially increase productivity in KM71H but most likely would not change or even reduce productivity in the Mut+ strains.

### 3.3 Comparison of *P. pastoris* and *S. cerevisiae* as expression hosts

**Figure 2:**
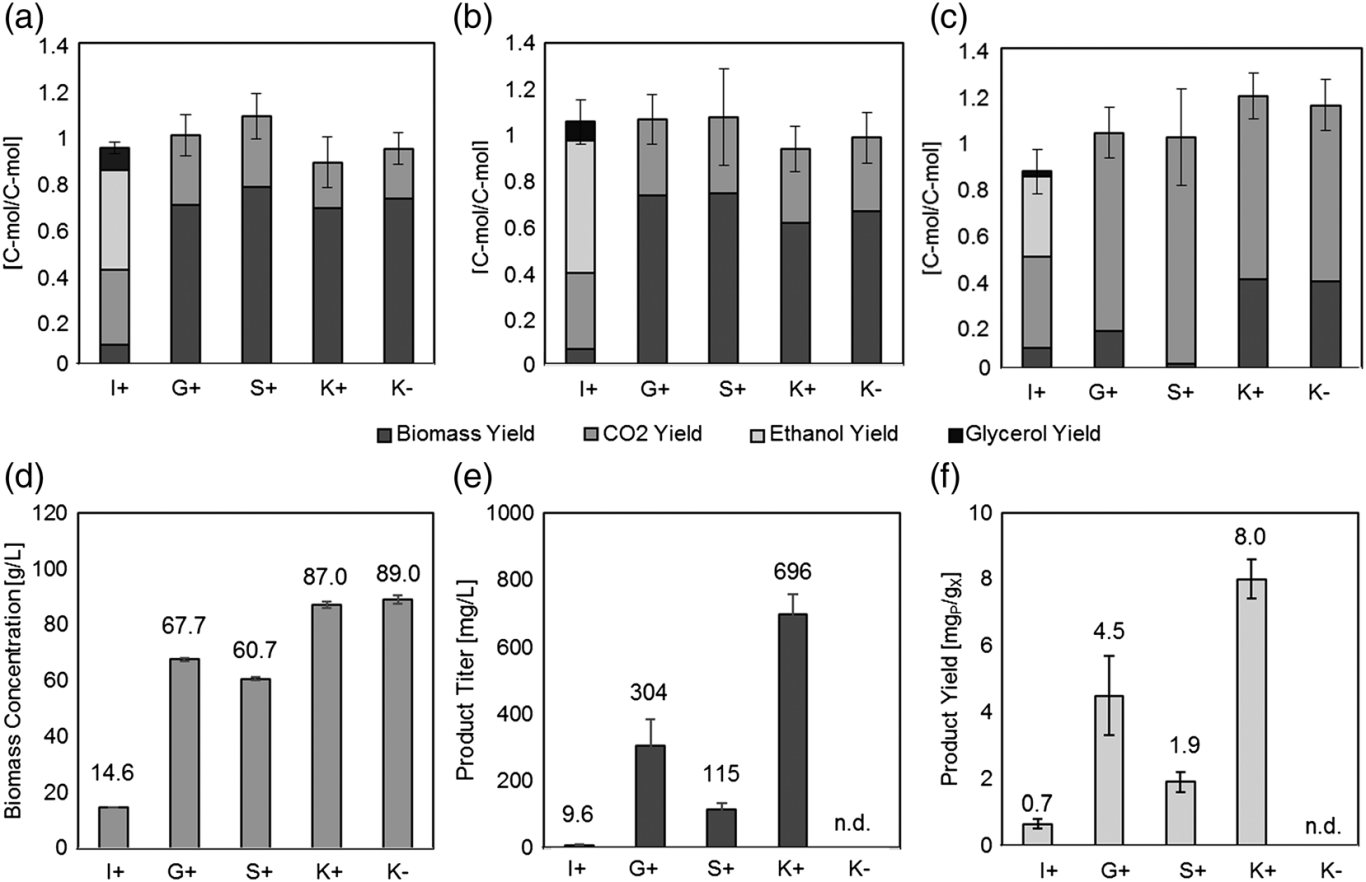
**Comparison of the different yeast strains regarding physiology and productivity**. Yields during the batch phase (A), fed‐batch phase (B) and induced fed‐batch phase (C) are shown as well as the obtained dry cell weight concentrations (D), product titres (E) and product yields (F) at the end of the cultivations.”I” indicates *S.* *cerevisiae* strain INVSc, “G” *P.* *pastoris* strain GS115, “S” *P.* *pastoris* strain SMD1168H and “K” *P.pastoris* strain KM71H. “+” is written for strains expressing CH3H, “‐“for empty strains. For (A), (B) and (C) only the errors on the C‐balances (calculated by error propagation) are shown for better readability. Errors on the respective yields can be found in Tables 2 and 3. For (D), (E), and (F) errors were calculated from at least three measurements. Differences in productivity were determined as significant at a 95 % confidence level

In Figure 2, the different hosts are compared in regard to their physiology and productivity.

Regarding physiological parameters in the cultivations of *P. pastoris* and *S. cerevisiae*, it is obvious that *P. pastoris* is favorable when it comes to conversion of C‐source into biomass as higher biomass concentrations can be achieved and the biomass yield during the batch and fed‐batch phase is clearly elevated compared with *S. cerevisiae* (2A‐D).

Regarding productivity it can be stated that even the lowest product yield in *P. pastoris* found in strain SMD1168H, is more than twice as high as the product yield found in *S. cerevisiae* (Figure 2F). The obtained results for expression of CH3H, strongly argue for employment of *P. pastoris* as expression hosts for cytochrome P450s, since both, biomass concentrations and specific product yields were higher in comparison with *S. cerevisiae.*

In general the cytochrome P450 yield found in our study is higher compared with values reported so far in literature which range around 50 to 400 pmol/mg protein for cytochrome P450 expression in yeasts (Hausjell, Halbwirth, & Spadiut, 2018). The product yield we found of 8.0 mg CH3H per gram dry cell weight corresponds to 600 pmol/mg protein, which is 1.5‐times as high as the highest expression yield reported (400 pmol/mg (Haudenschild, Schalk, Karp, & Croteau, 2000)). Nevertheless, these results are hard to compare as recombinant production of cytochrome P450s has not often been studied in controlled bioreactor cultivations but rather in shake‐flask experiments.

Although the product yield is already remarkably high, there could be room for improvement by optimization of other process parameters such as temperature, growth rate or induction time, which were kept at the same level in this study for all strains to allow comparability.

Finally, in spite of these promising production results, it has to be kept in mind that the protein needs to be purified for structure‐function elucidation. This can be quite challenging as the protein has to be extracted out of the membrane by detergents while retaining its native conformation (Prive, 2007). Usually numerous different conditions, including various detergents and concentrations thereof, buffer compositions, incubation times and temperatures as well as protein concentrations are screened to find the most advantageous method (Newby et al., 2009). Considering the number of experiments needed, the high biomass and product yield found in the CH3H expressing KM71H strain provide an optimal starting point.

## 4 CONCLUSION

Summing up, we recommend the yeast *P. pastoris* for the production of cytochrome P450s. It is Crabtree negative and thus allows more straightforward cultivations up to much higher biomass concentrations. Among the tested *P. pastoris* strains, the MutS phenotypic KM71H clearly was the best suited for production of CH3H. We believe that this host could be suitable for the expression of many eukaryotic, especially plant‐derived, cytochrome P450s as it combines high specific product yields together with the capability of cultivations to high cell densities. It thus provides optimal prerequisites for either biotransformation by cytochrome P450 enzymes or establishment of purification procedures for the protein allowing structure function elucidation.

## CONFLICT OF INTERESTS

The authors declare that they have no conflict of interests.

## APPENDIX 1

**Table A1:** Additional file 1: Table of primers used for cloning

Nr.	Name of Primer	Primer Sequence 5‘ – 3‘
1	DvCH3H_SF	AGCGGCTCTTCAATGGCTATTTTGCCTTTGTTGCTTT
2	R_DvCH3H_SR	AGCGGCTCTTCTCCCGGAACCTCTGGACTCGTAAA
3	F_pPICZA_TEV	cttccaatcgCATCATCATCATCATCATTGAGTTTTAGCCT TAGACATGACTGTTCCTCAG
4	R_pPICZA_TEV	tagaggttctcGGGCCCAAGCTGGCGGCC
